# Supplementary material for: Are carbon emissions trading and green financial instruments synergistic? -Comprehensive quantitative research based on content analysis
Source: PLoS One. 2024 Mar 7;19(3):e0298601. doi: 10.1371/journal.pone.0298601 (PMC10919601; doi:10.1371/journal.pone.0298601)
Supplement: S1 Data — (ZIP) [file pone.0298601.s001.zip › Data/地级市Z_F工作报告.zip!/║╙─╧/╨┬╧τ╩╨/└┤╫╘╣1⁄2╓┌║┼í╛┬φ┐╦╩2╛▌═°í┐_2012.docx]

各位代表：

现在，我代表市人民政府向大会作工作报告，请予审议，并请各位政协委员提出意见。

一、2011年工作回顾

2011年，市人民政府在省委、省政府和市委的坚强领导下，在市人大、市政协监督支持下，深入贯彻落实科学发展观，带领全市人民锐意进取，务实重干，加快发展，圆满完成了市十一届人大三次会议确定的各项目标任务。

（一）发展速度持续加快。实现地区生产总值1501亿元，在全省晋升两个位次，位居全省第7位，增长14.6%，增幅居全省第3位。财政一般预算收入90.7亿元，位居全省第4位，增长28.8%，税占比达到74.4%，同比提高1.1个百分点。固定资产投资1129.5亿元，增长29.3%，总量和增速均位居全省第4位。规模以上工业增加值735.6亿元，增长24%，增速位居全省第1位。社会消费品零售总额461.6亿元，增长18.9%，增速位居全省第3位。金融机构存款余额1306.3亿元，位居全省第4位；贷款余额781.8亿元，位居全省第5位。城镇居民人均可支配收入17988元，增长14.2%；农民人均纯收入7533元，增长20.7%，高于全省1.1个百分点，是近年来增长最快的一年。

（二）发展后劲持续增强。招商引资成效明显。实际利用外资5.3亿美元，位居全省第4位，增长61.3%；实际利用市外内资300.5亿元，位居全省第5位，增长34.1%。新签约央企合作项目5个，累计与20余家央企建立合作关系。新乡口岸作业区综合服务区建设正式启动，完成进出口总额16.4亿美元，增长44.8%，其中出口9.1亿美元，增长30.5%。项目质量水平提高。新开工亿元以上项目258个，同比增加90个。亿元以上项目占全市项目投资的48.5%，提高26.5个百分点，其中工业投资比重达到71.5%。赵固二矿、中粮（新乡）现代粮食产业化基地等66个省市重点项目竣工投产。要素保障能力增强。获批新增建设用地4.1万亩，是年度计划指标的8.7倍；其中报国务院批准中心城区用地8022亩，位居全省第3位。新增贷款和利用资本市场直接融资达到200亿元。成功发行12亿元城投债券。新引进交通银行新乡分行、辉县珠江村镇银行两家金融机构。3家企业向证监会上报首发申请，省重点上市后备企业15家。产业集聚区载体功能提升。产业集聚区（专业园区）建成区面积达到146平方公里，规模以上工业增加值556亿元，占全市工业增加值的75.6%。长垣县产业集聚区进入全省十强产业集聚区，高新技术产业集聚区、电源产业集聚区进入全省十快产业集聚区，进入全省十强、十快产业集聚区数量位居全省第1位。平原新区完成固定资产投资30.1亿元，50平方公里起步区基础设施建设基本完备。

（三）发展方式持续转变。三次产业比重由上年的13.2∶57.7∶29.1调整为12.5∶61∶26.5 ，工业化率提高3.7个百分点。新型工业化快速推进。规模以上工业企业销售收入2971.7亿元，增长40.4%，高于全省4.5个百分点，销售收入超亿元工业企业增加91家。42个工业项目列入全省重点产业年度行动计划，占全省项目总数的十分之一。纯电动出租车示范运营正式开展。新兴产业实现增加值138.8亿元，增长28.3%；高技术产业实现增加值82亿元，增长26.3%，增速位居全省前列。被列入国家老工业基地调整改造规划范围，工业结构调整将得到更多的政策支持。现代农业加快发展。完成南水北调渠首及沿线土地整治重大项目和国家500亿公斤项目年度工程。34座引黄调蓄工程全部列入国家规划。建成高标准农田20万亩，改造中低产田18.5万亩，新增有效灌溉面积6万亩。粮食总产390.2万吨，实现“六连创”，长垣县小麦高产攻关田最高亩产达到788.2公斤，创全省新记录。高效经济作物种植面积达到350万亩。组建了新乡小麦产业集团，与茅台集团签订2万亩有机小麦合作协议。“三品一标”基地333万亩，占耕地面积的57%。地理标志农产品、绿色食品数量均位居全省第1位。畜禽规模化标准养殖比重达到86%。在全省率先完成丹江口库区第二批移民新村建设及移民搬迁任务。服务业稳步发展。浙江海宁皮革城、杭州四季青服装城、居然之家家居广场等大型商贸项目陆续建成开业，新乡商业的辐射范围进一步扩大。新乡银行入选省服务业重点企业。自主创新成效明显。设立了5000万元中小企业技术创新引导基金，新增国家级技术研发平台3家、国家级高新技术企业20家。成功创建国家新型电池及材料高新技术产业化基地、中国卫生材料生产基地。电池研究院筹建工作稳步推进，组建新乡医学院干细胞与生物治疗技术研究中心。金龙集团获省长质量奖。专利申请量实现新突破，达到2369件。荣获国家科技进步先进市。生态环境保护得到加强。10个重大节能项目建成投产，新增节能能力 20万吨标准煤。5条出境河流断面水质达到省里要求，城市集中饮用水源地水质稳定达标，城市环境空气质量持续改善。完成凤泉区遗留铬渣处置任务。实现林业生态建设16.9万亩。获得中国最佳生态宜居城市称号。

　　（四）城市形象持续提升。规划引领理念得到强化。加快完善规划体系，城市总体规划顺利通过部级联席会议评审，完成了122个乡镇、851个新型农村社区规划，达到了近期建设用地控制性详细规划的全覆盖。实施了“数字城管”二期工程，城乡一体化联网运行稳步推进，获得中国最佳城市管理奖。城市建设步伐加快。“三大”活动持续推进，大整治完成拆迁545万平方米；大绿化新增绿地面积71万平方米，顺利通过“国家园林城市”复查验收；大建设完成投资228亿元、实施项目712项，23个棚户区和20个城中村改造项目启动，完成拆迁237万平方米，棚户区安置房新开工8572套。石武高铁车站区域路网基本形成，新区核心区100个重点工程全部开工，机场迁建取得重要进展。交通基础设施建设成效显著。公路总里程达到12990公里，每百平方公里密度高于全省平均水平11个百分点，基本形成了以市区为中心、京港澳等干线公路为骨架的快速交通网络。在全省率先完成县域城乡公交一体化试点改造。代表全省并圆满完成迎接全国干线公路养护管理检查工作。被国家批准为以“城乡一体化制度建设”为主题的全国农村改革试验区。统筹城乡工作积极推进，城镇化率达到42.89%，高于全省2.3个百分点。52个示范社区基础设施逐步完善。全市新开工建设新型农村社区1017万平方米，新入住农户4.2万户；拆除旧宅面积2.2万亩，复垦或恢复生态1.2万亩。首批规划启动了19个农民创业园建设，促进农民实现就近就业。

（五）人民生活持续改善。直接用于就业、社保、教育、医疗卫生、文化、保障房建设等民生支出95.8亿元，增长48.8%。圆满完成省市承诺为民办理的实事。就业创业工作不断加强。城镇新增就业13.9万人，下岗失业人员再就业4.9万人，就业困难人员再就业1.8万人，转移农村劳动力127.4万人次。创业型城市创建积极推进，开展创业培训4120人，发放小额贷款4.4亿元，个体工商户达到12.8万户，私营企业达到2.1万家，从业人员292万人。社会保障水平持续提升。城镇企业基本养老保险参保71.5万人，为18.5万企业离退休人员发放养老金29.7亿元。城乡居民社会养老保险在全省率先实现全区域覆盖。城镇基本医疗保险实现市级统筹，参保136.3万人，医疗保险付费制度改革走在全国前列。城镇居民医保补助标准提高到每人每年200元。农民工参加工伤保险14.6万人。对9917户低保家庭实施临时价格补贴。新建成养（敬）老院9家。保障性住房开工建设35953套，其中，新建廉租住房18086套，占全省总量的五分之一，各类保障性住房均已完成实物工程量的50%以上。各类教育协调推进。拨付各类教育资金45.7亿元，各类教育经费得到较好保障。新成立26对联合校。新建（改扩建）公办幼儿园82所。改造校舍43万平方米。30余所职业学校与富士康集团签订校企合作协议。支持驻新高校发展，校地、校企合作扎实推进。医疗卫生事业不断进步。乡村两级医疗机构全面实施国家基本药物制度，累计建成标准化卫生室2782个，新农合参合率达到99.5%，位居全省第1位。公共文化服务体系建设逐步完善。文明创建和思想道德建设持续推进，群众文化活动和优秀文化产品日益丰富。新建乡镇综合文化站68个、农家书屋838个，市平原博物院、市图书馆、市群众艺术馆免费开放。新发展农村有线电视用户2万户。各项事业协调推进。新解决32.4万农村居民饮水安全和40个村近10万群众出行难问题。圆满完成省定5.13万贫困人口脱贫任务。不断加强民族宗教事务管理，民族关系、宗教关系和谐发展。驻新部队大力支持地方建设，拥军优属、拥政爱民工作取得新进展，实现全国双拥模范城“五连冠”。社会大局保持稳定。平安建设深入推进，矛盾纠纷排查调处、社会治安防控、安全生产、应急管理等机制不断完善，警务机制改革创新得到国务委员、公安部部长孟建柱的充分肯定，实现全省平安建设先进市“六连冠”，获得中国最佳平安城市称号。对口支援新疆哈密地区伊吾县工作扎实推进。新闻出版、体育、统计、外事、侨务、史志、档案、测绘、人防、防灾减灾等工作都取得了新的成绩。

（六）政府自身建设持续加强。开展了“三抓两促”活动，进一步完善了政府工作细则，规范会议和议事规则，梳理出了358项重要难点问题，集中处理攻坚，目前已解决349项。倡导务实重干，健全责任、有序、高效的工作推进机制，加强行政效能监察，各级各部门克难攻坚、狠抓落实的能力进一步提高。一年来，我市先后10余次在全国、全省重要会议上作典型发言，10余次全省专题现场会在我市召开，上级对我市相关工作给予了充分肯定。坚持依法行政，自觉接受人大法律监督和政协民主监督，累计办理人大政协议案、提案和建议668件，办结率100%。落实首问负责、服务承诺、限时办结等工作机制，提前5个月完成全年联审联批任务。精简行政审批事项148项。强化国有资产监管和审计监督，财政资金使用效益不断提高。着力构建“四位一体”的监督管理服务体系，其中公共资源交易中心累计交易项目6423项，从源头上预防腐败的体制机制不断完善。

各位代表！这些成绩的取得，归功于历届市领导班子的传承接力和全市人民的团结拼搏，得益于社会各界的大力支持和帮助。一个干事创业、团结和谐的新乡，一个自强不息、务实重干的新乡，一个日新月异、充满活力的新乡，正以昂扬向上的姿态展现在世人面前。在此，我代表市人民政府，向各位人大代表和政协委员，向全市各条战线上的广大工人、农民、知识分子、干部、驻新解放军指战员、武警官兵、公安干警及社会各界人士致以崇高的敬意！向所有关心、支持新乡发展的朋友表示衷心的感谢！

各位代表！我们也清醒地认识到，新乡仍然处于蓄势积累、爬坡上坎的关键时期，经济总量小、人均指标低、发展不够快等问题依然突出，晋位升级的任务十分艰巨；三次产业发展不够协调，县域发展不平衡，创新能力仍需加强，财政收入结构和质量有待进一步优化和提升，资源环境约束加剧，转变发展方式的任务十分艰巨；公共服务依然滞后，社会事业发展与群众需求的差距较大，上学、就医、住房改善任务繁重，就业社保压力较大，改善民生的任务十分艰巨；少数单位依法行政观念不强，一些干部和工作人员工作作风、办事效率仍需转变和提高，消极腐败现象依然存在，政府自身建设有待进一步加强。对于存在的这些问题，我们必须在今后的工作中着力加以解决。

　　二、2012年工作总体要求

今年工作的指导思想是：认真落实省九次党代会、省委经济工作会和市十次党代会精神，围绕科学发展主题和转变经济发展方式主线，以富民强市为中心任务，坚定不移地实施引资项目双带动战略，坚持走不以牺牲农业和粮食、生态和环境为代价的“三化”协调科学发展路子，打造中原经济区统筹城乡发展示范区、先进制造业基地、现代农业试验示范基地和高素质人力资源培育基地，加快结构调整，着力改革创新，更加注重民生改善，保持社会稳定，持续求进，务实发展，为全面建设幸福新乡、实现综合实力迈入全省前列、打造中原经济区强市奠定坚实基础。

经济社会发展预期目标是：生产总值增长12%，全社会固定资产投资增长20%左右，公共财政预算收入增长15%，社会消费品零售总额增长16%，城镇居民人均可支配收入、农民人均纯收入均增长10%，城镇登记失业率控制在4.6%以内，人口自然增长率控制在6.5‰以内，节能减排指标控制在省定目标之内。

各位代表！实现上述目标，虽然面临全球金融危机对实体经济冲击和我市结构调整必须付出代价的挑战，但更有国务院支持中原经济区建设和国家扶持实体经济发展强力带动的难得机遇。我们深知，机遇不会永远存在而是稍纵即逝，机遇不会唾手可得而需奋力拼抢。面对激烈的竞争态势，新乡发展不进则退、慢进亦退，我们没有懈怠的理由、没有彷徨的借口、没有陶醉的资本，必须抢抓机遇、再鼓干劲、乘势而上、跨越发展。我们坚信，有省委、省政府和市委的坚强领导，有市四大班子的团结协作，有全市干部群众的共同努力，有方方面面的大力支持，只要我们毫不动摇地坚持实践证明正确的发展思路，持之以恒地抓好各项重点工作落实，新乡发展一定能够实现更大跨越！

三、2012年重点工作

今年重点抓好以下七个方面的工作：

（一）加快新型城镇化，强化引领作用。优化城镇布局。按照“核心带动、轴带发展、节点提升、对接周边”的要求，构筑“一主、两副、五组团”现代城镇体系，坚持中心城市组团式发展、县城和重点镇内涵式发展、新型农村社区集聚式发展，推动城市发展由速度型向品质提升型转变，打造中原经济区城市群核心区北部支撑极。强化城乡规划和管理。坚持以科学的理念制定规划，以科学的规划引领城乡建设，维护规划的严肃性、权威性。编制中心城市、县城、重点镇、新型农村社区四级现代城镇体系全域规划，完成50个专项规划编制和城市总体规划报批工作。完成红线、绿线、黄线、紫线、蓝线和橙线“六线”控规制定，实现市、县建成区和重点镇主要街区控制性详细规划全覆盖。深化城市管理长效机制建设，确保省“数字城管”达标验收。严格依法规范出租车、摩的运营，强力实施火车站、汽车站等区域环境综合整治。市区完成30座垃圾中转站升级改造，新建公厕50座，加快推进城乡环卫一体化，确保通过全国卫生城市复检。强化中心城区建设。坚持“有机更新、均衡发展”。新区方面，积极推进以高铁车站区域为核心的第二商业中心板块、以大学生创业园和高新技术创新平台为核心的创新板块、以平原体育会展中心为核心的体育运动板块建设，将新区重点督导项目扩展到150个，加快平原文化艺术中心、新闻传媒中心等重点项目建设。旧城方面，加大老城区升级改造力度，强化城市西区基础设施和公益设施投入，机场搬迁上半年取得实质性进展，争取早日开工建设；市区实施15个城中村改造项目，完成拆迁80万平方米，开工建设50万平方米，竣工30万平方米，开工建设棚户区安置房5620套。道路方面，谋划老火车站至高铁车站的缓堵保畅快捷路建设；按照南环东段标准，谋划推进西环、北环和南环西段改造提升及环路绿道建设；抓好高铁车站广场区域交通零换乘及主要道路等基础设施建设；积极推进市区主要交通路口的渠化改造，充分挖掘现有交通设施潜力，提高通行效率，缓解城市交通压力；市区新建、续建、改造市政道路31条、38.5公里，宏力大道西牧村段7月底前完成拆迁任务，力争10月月底全线贯通。水系景观方面，实施赵定排疏通及景观整治、卫河市区段提升改造，谋划建设高铁站生态湖公园、人民西路公园和南湖公园，争取早日实现“渠相连、水常清、水常流”。强化县城建设。持续开展“新城杯”竞赛，引进高水平、有实力的投资者，加快旧城和城中村拆迁改造，扩大县城规模，提升建设品质。卫辉市、辉县市、新乡县、长垣县拆迁不低于40万平方米，原阳县不低于35万平方米，获嘉县、延津县、封丘县不低于25万平方米。推动辉县市、长垣县等基础条件较好的县城向中等城市规模发展。加快推进重点乡镇“三化”协调发展。坚持镇区开发与新型农村社区建设相结合，在40个重点乡镇的基础上，确定20个左右示范乡镇，支持其率先实现“三化”协调发展。支持孟庄、古固寨、唐庄等乡镇进行镇区开发试点建设。深化财税、金融、投资等体制改革，扩大重点乡镇管理权限。进一步加大新型农村社区建设力度。坚持把新型农村社区作为推进城乡一体化的切入点、统筹城乡发展的结合点和促进农村发展的增长点，充分发挥新型农村社区的战略基点作用，进一步加大推进力度，努力实现新突破。52个示范社区每个县（市）、区至少有一个社区完成建设任务，其余示范社区完成70%以上的建设入住和旧村拆迁复垦任务，同时继续推进200个重点社区基础设施和服务设施的完善工作。研究出台对已复垦土地进行申报验收和腾退土地使用管理的具体操作流程和办法，年底前实现累计拆迁4.5万亩，复耕2万亩。建设开放型城市群。以城乡交通体系建设为突破口，推进县（市）之间交通一体、产业链接、服务共享、生态共建，打造具有较强竞争力的开放型城市群。加快新晋高速、鹤辉高速、京港澳高速立交和长济高速东明黄河大桥等项目建设。前期推进郑焦高速东延至大广高速、新晋高速块村营至平原新区建设路网规划，打造环绕市区、县（市）互通的高速路网。实施公交优先发展战略，加快公交场站建设，优化公交线路布局，逐步实现城乡公交全覆盖。

　　（二）加快新型工业化，提升核心竞争力。实施工业新增长点工程，以新型电池与电动车（含专用车与汽车零部件）、生物与新医药、现代煤化工及煤电、制冷及电子信息、特色装备制造、传统优势产业（食品、造纸、纺织、建材等）为重点，力争用5年左右的时间，使六大产业都达到千亿规模，构建具有持续竞争力的现代产业体系，打造中原经济区新型工业化先行市。做大做强产业。新型电池与电动车产业方面，重点抓好环宇集团5亿安时汽车用锂离子电池、新航集团高科技产业园等项目的实施，确保环宇集团2万辆电动轿车及2000辆电动大巴动力电池、中科公司1亿平方米锂电隔膜等项目竣工投产。抓好电动车示范运营，完善新型电池与电动车产业链条。现代煤化工及煤电产业方面，重点抓好中科化工整体搬迁及180万吨甲醇深加工一体化、心连心化肥年产80万吨尿素、新中益2×60万千瓦火电机组、渠东热电2×33万千瓦热电机组等项目的实施，加快推进中联煤层气开发、煤炭物流中心等重点项目建设，力争华源护神1000万平方米防寒透湿复合材料、银金达1.2万吨热收缩膜等项目建成投产。完善乙二醇-聚酯-下游产品、甲醇-烯烃-下游产品产业链。生物与新医药产业方面，重点做好双鹭生物医药产业园、拓新生化200吨系列阿糖核苷等项目实施。完善生物技术药物、化学成品药、现代中药等主导产业链。制冷及电子信息产业方面，重点做好中兵通信科技产业园、中国电波科技城、新航集团120万台汽车空调压缩机等项目的实施。深入研究制冷产业发展，着力引进终端企业，带动配套产业和制冷材料产业发展，努力恢复制冷产业名片。特色装备制造产业方面，重点做好长兴精工10万吨装备钢结构、锦隆重工1500套矿用救生舱等项目的实施。培育壮大新能源装备和节能环保装备等产业，做强专用设备等亮点产品。传统优势产业方面，重点抓好百威英博100万吨啤酒、白鹭化纤1万吨新型溶剂纺纤维素、北新建材6000万平方米石膏板生产线等项目的实施，争取新亚集团40万吨食品卡纸等重点项目早日竣工投产。坚持用高新技术和先进适用技术改造提升传统产业，提升核心竞争力。打造产业基地。紧紧抓住国务院《指导意见》支持河南重点产业基地建设的重要机遇，加快信息化与工业化深度融合，推动新乡老工业基地加快改造，依托优势产业和产业集群，打造中国电池研发和产业基地、汽车零部件生产基地、特色装备制造基地、现代煤化工基地、生物医药基地、制冷及电子信息产业基地、现代物流基地和物联网基地。强力推进企业上市。积极实施企业上市引导计划，提高企业上市积极性；加强上市后备企业培育，推进新亚纸业、拓新生化、高远公路等企业尽快进入辅导；切实抓好金龙铜管、日升数控、天丰节能板材等3家已申报在审企业与证监会的沟通，争取年内3家到4家企业发行上市。培植壮大龙头企业。鼓励龙头企业多渠道筹集资金，充分利用产业联盟、资产重组、控股参股、兼并租赁等多种方式扩大企业规模。引导央企和国内外行业龙头企业参与我市产业重组，争取在新乡扩大规模，建设区域总部、研发中心、营销中心和生产基地。强化要素保障，支持市规模效益50强企业和30户高成长性企业快速发展壮大，争取更多企业进入省“双百”企业行列。深入推进质量立市。扶持优势企业争创政府质量奖，力争再创1个到2个省长质量奖，创建省名牌产品20个。大力实施商标带动战略，选择1家到两家企业进行重点帮扶，支持争创中国驰名商标，推荐申报10个河南省著名商标。强化自主创新。始终把创新作为新乡的灵魂，把突破和掌握核心技术作为产业可持续发展的关键。按照国家级工程中心标准建成电池研究院，筹建煤化工技术研究院，争取中科院煤炭综合利用示范基地、生物科研基地落户新乡。推动河南动力电源及关键材料工程技术研究中心、新乡医学院干细胞与生物治疗技术研究中心等建设与发展。实施好省重大科技专项，新建市级工程技术研究中心、重点实验室20家。专利申请量保持全省前列，发明专利比重达到20%以上。用好中小企业技术创新引导基金，扶持科技型中小企业发展。

（三）加快新型农业现代化，打造特色和亮点。提高农业综合生产能力。强化耕地保护和土地开发整理，加大农田水利基础设施投入和农业综合开发力度，抓好国家千亿斤项目工程、南水北调渠首及沿线土地整治重大项目、省高标准粮田“百千万”工程和引黄调蓄工程实施，新增有效灌溉面积2万亩、节水灌溉面积10万亩。着力开展小麦、玉米、水稻三大粮食作物高产创建活动，提高单产，完善农业气象服务体系建设，争创全省粮食生产核心区优质高产示范区，确保粮食生产能力稳定在80亿斤以上。发展高效农业。引导市区近郊乡镇发展高端设施农业，大力发展食用菌、蔬菜、花卉等高效经济作物。积极推动品牌农业进行优化重组，壮大企业规模，做大做强“新乡小麦产业集团”，打造“中国第一麦”品牌；组建“原阳大米产业集团”，叫响“中国第一米”品牌。扶持封丘金银花、获嘉鲜切花、长垣食材等产业不断壮大。新增市级以上名牌农产品15个。提高现代畜牧业水平。开展标准化规模养殖，新增100个标准化示范规模养殖场（小区），奶牛存栏达到9万头以上。完善基层动物防疫体系，推进区域性动物防疫检疫中心站建设，标准化建站率达到50%以上。加快农业产业化。培育发展农业产业化龙头企业，促进农民专业合作社规范发展，新增市级以上龙头企业10家，新发展农民专业合作社200家。大力发展辉县生猪及肉制品、原阳奶制品、长垣高效蔬菜等十大产业集群。打造农业科技产业化先行市。深入贯彻落实“中央一号文件”精神，有效利用新乡独有的农业科技立体支撑优势，充分发挥中国科学院、中国农科院、省农科院、市农科院等四大现代农业科研基地引领示范作用，加快河南师范大学、河南科技学院及市级以上14个科技创新平台和工程技术研究中心建设，争取更多的科研项目在新乡布局和转化，不断增强我市现代农业的竞争力。

　　（四）加快城乡统筹和农村综合改革，努力实现新突破。加快推进全国农村改革试验区建设。完善总体实施方案，尽快进行项目布点，力争用3到5年时间，在粮食安全、土地集约节约利用、产权制度改革、社会管理模式完善等重点领域和关键环节取得突破，形成一套规范化制度体系，努力为全国、全省探索路子、积累经验。积极探索城乡一体的社会管理机制。进一步完善户籍管理制度，制定配套政策，有序推进农村人口向城镇和新型农村社区转移。探索建立与城乡一体化发展相适应的公共服务和社会保障机制。探索建立新型农村社区党总支委员会、社区服务管理委员会、社区居民代表委员会、社区居民事务监督委员会“四位一体”的农村社区组织机构，促进农村社区融合。积极探索农村土地集约节约利用的新机制。开展人地挂钩试点，建立城乡统一的土地市场，试验开展建设用地指标交易和集体建设用地使用权流转，促进城市资本反哺农村，让农民分享土地增值收益。组织开展农村集体产权股份合作制改革，在全市52个示范社区开展被整合村土地、集体资产、债权债务管理试点。加快产业集聚区建设。产业集聚区（专业园区）完成固定资产投资850亿元，规模以上工业企业实现主营收入3000亿元，从业人员新增5万人以上，力争在全省排名中晋位升级。坚持“规划领先、产业先进、低碳生态、城乡一体”的建设理念，强力推进平原新区基础设施和公共设施建设，强化招商引资，争取早日出形象、成规模。推进高新技术产业开发区“两化融合试验区”建设，加快新能电动车、新航集团大飞机零部件研发生产等项目建设进度，确保华兰生物血液制品、娃哈哈7期等项目竣工投产。以打造城市新组团为目标，提高新乡工业园区建设发展水平，力争与中兵集团、中航集团、双鹭药业等行业龙头企业的合作取得新的进展。争取高新技术开发区、新乡工业园区早日创建成国家级高新技术开发区和经济技术开发区。着力培育壮大卫辉市食品加工和新型建材、辉县市特色装备制造、新乡县生物医药和振动机械、获嘉县现代煤化工、原阳县食品加工和汽车零部件、延津县食品加工和装备制造、封丘县纺织服装、长垣县起重装备等优势产业集群。切实抓好扶贫开发。着力构建专项扶贫、行业扶贫、社会扶贫三位一体的“大扶贫”格局，把沿黄地区、太行山区、黄河故道区、贫困革命老区与特殊困难地区作为主战场，以整村推进和扶贫搬迁为重点，完成50个扶贫开发重点村整村推进建设和260户、1000人深石山区贫困人口搬迁任务。抓好1.7万丹江口库区移民帮扶工作。确保贫困地区农民人均纯收入增幅明显高于全市平均水平。

（五）加快招商引资和项目建设，增强发展后劲。强力实施“引资项目”双带动战略，坚持把项目作为经济工作的综合性抓手，把招商引资作为项目建设的主渠道。大力开展产业链和产业集群招商。围绕优势支柱产业，以产业链整合提升为重点，逐行业论证梳理，绘制产业链条图谱，针对薄弱环节和产业链缺项开展招商，提升关键环节，完善配套能力，促进产业结构优化升级。深入摸排沿海产业集群及其转移动向，根据自身优势，适时派专人赴产业集中、特色鲜明的地区长期驻地、蹲点招商，吸引沿海电子信息、食品等产业集群式转移。大力开展以商招商。建立完善激励机制，调动市内外中介机构和自然人的招商积极性，突出招大引强、二次招商。抓好与央企对接合作，深入研究中兵、中航、中粮等已落地新乡的大型企业集团发展战略和投资方向，制定后续招商项目单子；争取中兵集团特种汽车、中航集团火车转向支架、中材集团特色装备制造等重大项目落地。着力打造开放高地。抓好“招商引资项目落地年”活动，全年实际利用外资6亿美元以上，市外内资360亿元以上，引资总量继续保持全省前列。抓好口岸作业区建设及综合保税区申建工作，争取海关项目上半年完成主体施工，做好综保区规划和土地利用规划、城市规划的衔接工作，积极争取国家部委支持，为最终获得国务院批准打下基础。强力推进重点项目建设。实施亿元以上项目772个，完成投资791.5亿元，确保77个省重点项目和209个市重点项目按计划完成建设任务。把提高在建项目建设进度和投资强度作为重中之重，加快中航高科技产业园二期、胜华电缆工业园一期、欧玛汽配工业园等50个总投资5亿元以上重大工业项目建设；确保豫飞重工新型工业化装备产业园、卫华重型装备产业园等213个亿元以上项目竣工投产；确保中航新飞专用汽车产业园、科隆集团新能源材料产业园等326个亿元以上项目开工建设；谋划推进重大支撑项目，加大现代煤化工基地、郑新城际铁路、中原经济区大学科技园等80个总投资2800亿元的“十二五”重大支撑项目推进力度。健全工作推进机制。坚持招商引资党政“一把手”工程，县（市）、区党政一把手每季度以书面形式向市委、市政府进行工作汇报；定期在媒体公布各地招商引资和重点引资项目进展情况；实施半年度和年度考评末位警示。健全重大项目周例会、联审联批制度，提高重大项目前期手续审批、备案效率。合理调度土地、环境容量、资金等要素，提高项目合同履约率、开工率和资金到位率。

（六）加快服务业发展，优化产业结构。打造区域性商贸中心城市。合理调整城区商业布局，完善服务业功能，做好台湾百脑汇数码广场、牧野湖下游卫河两岸休闲文化娱乐城（街）等商贸重点项目的跟踪推进，谋划城郊大型购物中心。以南环、小店、平原新区等综合物流园区为重点，加快传统运输业向现代物流业转变。以中粮集团、中原经济区生物医药产业园、新月复线煤炭中转站等项目为重点，推进物流业与制造业、现代农业联动发展。继续实施“新网工程”，构建农村现代流通网络。大力发展旅游业。整合提升南太行旅游资源，加快南太行旅游环路建设，发展牧野文化游，开发沿黄生态游，利用好海宁皮革城、杭州四季青服装城的购物游，推进旅游业由观光型向观光、休闲度假、商务等综合型转变。抓好旅游宣传、游客服务中心建设、境外旅游市场开拓等工作。实现综合旅游收入、接待国内游客人数分别增长15%和10%，力争入境游客达到5万人次。积极发展金融服务业。加强银企对接，努力提高银行存贷比，促进信贷投放，全年金融机构新增贷款90亿元以上。着力金融产品和服务创新，继续加大信托、中小企业集合债券等工作推进力度，实现银行间市场直接融资20亿元以上。积极引进域外金融机构，推动中信银行、郑州银行在我市设立分支机构，力争设立一到两家村镇银行，推动新乡银行增资扩股。进一步整顿和规范担保公司，打击非法集资行为，防范金融风险。推动牧野文化大发展、大繁荣。坚持把文化作为推动经济社会发展的重要杠杆，作为社会文明进步的重要标志，加快文化产业发展，强化公共文化体系建设，培育打造文化精品。争取千盛银河影城、中影集团3D影院项目落地，推进卫辉比干文化园、关帝庙历史文化街、卫河市区段文化元素提升等重点项目建设，建成长垣县中国烹饪文化博物馆。全力推进大运河申报世界物质文化遗产工作。完成30个新型农村社区文化大院建设。新发展农村有线电视用户2.5万户。文化产业增加值增长15%以上。

　　（七）加快幸福新乡建设，保障和改善民生。全面落实幸福新乡建设总体部署，大力实施经济支撑、民生保障、安全建设、文化建设、宜居环境等五大工程，确保完成省市承诺的实事办理工作，不断提升群众幸福感指数。做好创业就业工作。鼓励创业就业，以创业带动就业，城镇新增就业8.1万人；完善基层公共就业服务平台，抓好全民技能振兴工程实施，强化农民（移民）劳动技能培训，完成农村劳动力转移就业132万人次，打造创业型城市。完善工资共决机制，构建和谐劳动关系。健全社会保障体系。全面推进城乡居民社会养老保险，提高企业退休人员基本养老金并按时足额发放。完善城镇基本医疗、工伤保险市级统筹机制，扩大城镇基本医疗保险门诊重症慢性病病种范围。加强社会保障服务能力建设。建立低收入群体失业金补贴与物价上涨联动机制，适时提高失业金标准。落实用地和资金保障，开工建设31822套保障性住房。完善城乡救助体系，大力发展社会福利和慈善事业，积极推进居家养老服务。促进教育均衡发展。认真落实校市合作方案，抓好校企科研项目合作、高校优秀专家到企业挂职、大型检测设备及科研平台共享等19项具体工作落实，充分发挥高校的支撑作用。大力发展职业教育，全力推进职业教育管理体制机制改革，加快职业教育园区建设，启动新乡幼儿师范学校和新乡卫校新校区建设，支持两校升专。落实教育经费“三个增长”政策，促进义务教育均衡发展，抓好校安工程3年攻坚收尾和加固项目的实施工作，扩大“联合校”实施范围，调整优化城乡中小学布局，改造农村义务教育薄弱学校，支持民办教育发展。加强校园安全管理，严防重大责任事故发生。提高医疗卫生保障水平。完善新农合制度，筹资标准提高到290元，补偿封顶线提高到15万元，扩大重大疾病保障范围，按病种付费种类由30个扩大到65个。推行临床路径管理，推进支付方式改革，控制抗生素使用和药占比。区域卫生信息平台投入使用，实现“一卡通”和出院直补。为困难群众减免医疗费用1000万元，争取卫生部“健康快车”再次停靠新乡。开工建设新区三级甲等医院、市中心医院门诊楼、市一院全科医生培训大楼，确保市二院综合病房大楼投入使用。建设乡镇卫生院20个，全面完成行政村标准化卫生室建设任务。规范医疗秩序，构建和谐医患关系。加强食品药品安全监管。完善推广“飞测”制度，建立健全食品安全检测体系，严厉打击、从严查处食品非法添加剂和制售假劣食品药品等违法犯罪行为，确保食品药品安全。强化节能减排和生态环境保护。严格落实节能减排目标责任制，综合运用建设项目总量替代、排污许可证发放等措施，淘汰落后产能，发展循环经济。确保孟庄、贾屯、平原新区污水处理设施建成投运，现有污水处理厂升级改造9月月底前完成。完成省政府下达的出境断面水质控制目标，城市集中饮用水源地取水水质达标率100%。开展凤泉区遗留铬污染物生态恢复。完成48个农村环境连片综合整治项目。完成禁养区内的22家养殖企业关闭或搬迁。实施林业生态建设工程9万亩，确保“全国绿化模范城市”创建成功。建设运动城市。做好市区公共体育设施规划，推进体育场馆和设施建设改造，在社区等公共场所配备健身器材。按照建设、管理、运营一体化的原则，加快推进平原体育会展中心建设。积极开展全民健身活动，开好全市第十届运动会。大力发展其他事业。再解决20万农村居民的饮水安全问题。严格执行现行生育政策，稳定低生育水平，综合治理出生人口性别比偏高问题，引导人口合理分布。积极开展法律援助，依法保护妇女、未成年人、残疾人和老龄人的合法权益。抓好未成年人思想道德建设，广泛开展“爱我家园、共建文明幸福新乡”活动。大力支持驻新部队建设，抓好退伍安置和民兵预备役工作，巩固军政军民团结，继续开展双拥模范城创建活动。深入开展民族团结进步先进市创建和宗教创先活动，推动少数民族聚居地区经济发展、社会进步和文化繁荣。高度重视和发挥好工会、共青团、妇联、文联、侨联、社科联、残联等人民团体的桥梁纽带作用。做好新闻出版、统计、史志、外事、侨务、档案、测绘、人防、防灾减灾等工作，促进各项社会事业全面发展。

四、狠抓政府自身建设

坚持用领导方式转变加快发展方式转变，认真落实“三具两基一抓手”，深入推进“两转两提”，持续开展“三抓两促”，建设“依法行政、务实为民、廉洁高效”的服务型政府。

（一）坚持依法行政，认真履行职责。严格执行人大及其常委会的决议决定，依法接受其法律和工作监督，主动接受人民政协的民主监督，支持政协参政议政，认真办理人大代表建议和政协提案。广泛接受社会公众和新闻舆论监督。深化行政审批制度改革，健全政务服务体系。完善执法程序，规范执法行为。加强行政复议工作，强化行政执法责任和过错追究。健全行政监督体系，加强审计监督、行政监察和行政层级监督，坚决纠正行政不作为和乱作为。健全专家论证、技术咨询、风险评估以及听证、公示等制度，促进决策科学化、民主化、规范化。

（二）创新社会管理，转变政府职能。健全党委领导、政府负责、社会协同、公众参与的社会管理格局。拓宽政务信息、行政审批、公共资源交易等政务服务公开的领域。推进社会诚信体系建设，打造诚信新乡。完善基层社会管理和服务体系，不断强化城乡社区自治和服务功能。创新流动人口、特殊人群、移民村、非公有制经济组织和社会组织、虚拟社会的管理与服务。强化社会矛盾隐患排查和风险评估，加强和改进信访工作，完善社会矛盾动态管理机制，构建人民调解、行政调解、司法调解联动的大调解工作体系。深化事业单位人事制度、国有资产监管、医疗卫生、文化体制、林权、水利等各领域的改革。严格安全生产责任制，强化治安防控体系建设，加强现代指挥体系及实战警务机制建设，再创“全国社会治安综合治理优秀市”，争创“长安”杯。健全预防预警和应急处置体系，确保社会大局和谐稳定。

　　（三）强化责任意识，加强执行力建设。各级领导干部要带着责任去工作，在其位、谋其政、负其责、尽其心，认真负责地抓好各项重点工作落实。要形成工作即时报、即时批、即时办、即时干的快速推进机制，以目标确定进度，以时间倒逼程序，做到重点工作随时过问、随时推进、随时督查。要将重点工作落实到具体项目和人员，做到问题在一线解决、工作在一线落实。要强化责任追究和行政效能监察，做到各级各部门工作与全市中心工作思想同心、目标同向、推进同步，强力打造“信念坚定、忠诚履职、直面矛盾、务实善干、一抓到底、运转高效”的干部队伍形象。

（四）坚持务实为民，严格廉洁从政。高度关注困难群体的疾苦，认真落实惠民富民的政策措施，坚决纠正损害群众利益的不正之风。严格执行党风廉政建设责任制和领导干部廉洁自律各项规定，围绕“四位一体”的监督服务体系建设，切实规范资源开发、行政审批、资金安排、非税收入监管、公共资源交易等管理，探索从源头上、机制上防止腐败的有效措施。坚持勤俭节约，严格财经纪律，严控一般性支出，坚决抵制各种奢侈浪费现象。各级领导干部要常修为政之德、常思贪欲之害、常怀律己之心，不断增强拒腐防变能力。

各位代表！建设中原经济区强市的美好前景激励着我们，570万牧野儿女的热切企盼鞭策着我们，让我们紧密团结在以胡锦涛同志为总书记的党中央周围，深入贯彻落实科学发展观，奋发图强，克难攻坚，创先争优，谱写新乡跨越发展的新篇章，以优异的成绩迎接党的十八大胜利召开！
